# FXR and Nrf2 Mediate the Effect of Carbon Ion Irradiation on Drug Transporter OATP1A2 in Cancer Cells

**DOI:** 10.3390/biology15201886

**Published:** 2026-10-19

**Authors:** Zichong Li, Ziqi Zhang, Zhixing Li, Jiajian Yuan, Yarong Du, Mei Hong

**Affiliations:** 1 Guangdong Laboratory for Lingnan Modern Agriculture, College of Life Sciences, South China Agricultural University, Guangzhou 510642, China; 2 Institute of Modern Physics, Chinese Academy of Sciences, Lanzhou 730000, China; 3 Guangdong Provincial Key Laboratory for the Development Biology and Environmental Adaptation of Agricultural Organisms, South China Agricultural University, Guangzhou 510642, China

**Keywords:** OATP1A2, carbon ions, FXR, Nrf2, chemoradiotherapy

## Abstract

Organic anion transporting polypeptides (OATPs, gene symbol *SLCO*) are recognized as key factors in the absorption, distribution, and excretion of a wide range of endogenous and xenobiotic compounds, including many anti-neoplastic drugs. Several OATPs, such as OATP1A2, OATP1B1, and OATP1B3, have been found to be expressed in tumor cells and/or tissues, making them potential therapeutic targets for chemotherapy. Previous studies in our laboratory demonstrated that both X-ray and carbon ion irradiation induced the expression of OATP1A2 in the breast cancer cell line MCF7, resulting in enhanced killing effects of methotrexate, 5-fluorouracil, and paclitaxel following irradiation. In the present study, we evaluated the effect of carbon ions on the expression of OATP1A2, OATP1B1, and OATP1B3 in different cell lines (HCT116, HepG2, A549, and MCF7). It was found that only HepG2 and MCF7 cells exhibited an upregulation of OATP1A2 after irradiation. Mechanistically, carbon ions induced the expression of FXR and Nrf2, both of which specifically bind to the upstream regulatory sequence of *SLCO1A2*, the encoding gene for OATP1A2. Modulators of these transcription factors altered the induction of OATP1A2 following irradiation. Additionally, combined treatment of these modulators, methotrexate, and carbon ion irradiation resulted in a more significant killing effect on these cancer cells.

## Simple Summary

Organic anion transporting polypeptides (OATPs, encoded by *SLCO* genes) play critical roles in the disposition of endogenous substances and xenobiotics, including numerous anticancer drugs. Several OATPs—notably OATP1A2, OATP1B1, and OATP1B3—are expressed in tumor tissues, positioning them as promising chemotherapeutic targets. Our prior work showed that both X-ray and carbon ion irradiation upregulate OATP1A2 in MCF7 breast cancer cells, enhancing the cytotoxicity of anti-neoplastic agents post-irradiation. In this study, we examined carbon ion effects on OATP1A2, OATP1B1, and OATP1B3 expression across several cancer cell lines. Notably, only HepG2 and MCF7 cells displayed increased OATP1A2 level following irradiation. Mechanistically, carbon ions elevate the level of transcription factors FXR and Nrf2, which bind specifically to the *SLCO1A2* upstream regulatory region. Pharmacological modulation of these factors altered irradiation-triggered OATP1A2 induction. Furthermore, combining these modulators with methotrexate and carbon ion irradiation produced significantly enhanced cancer cell killing, suggesting a novel strategy to improve radiotherapy outcomes via OATP-mediated drug uptake.

## 1. Introduction

Organic anion transporting polypeptides (OATPs, gene symbol *SLCO*) are membrane transporters recognized as critical factors in the absorption, distribution, and excretion of a wide range of endogenous and xenobiotic compounds [1]. The substrates for OATPs primarily consist of amphipathic organic molecules, including bile salts, hormones and their conjugates, toxins, and various drugs. Eleven functional OATPs have been described in humans to date, namely OATP1A2, 1B1, 1B3, 1C1, 2A1, 2B1, 3A1, 4A1, 4C1, 5A1, and 6A1 [2,3,4]. Some are distributed broadly across multiple tissues, while others are confined to particular organs. For instance, OATP1B1 and 1B3 are essentially liver-specific, while OATP1A2—recognized as the first human OATP to be characterized [5]—is localized in the brain, liver, kidney, and intestine [6]. Recent studies have shown that OATPs can be differentially distributed in tumor cells and tissues. For example, OATP1A2, along with OATP1B1 and OATP1B3, have been found to be highly expressed in pancreatic adenocarcinoma [7]. OATP1A2 level was elevated under androgen-depleted conditions, suggesting a link to adaptive cell growth in prostate cancer cells [8]. Furthermore, it has been demonstrated that OATPs, particularly OATP1A2, OATP1B1, and OATP1B3, can transport various anticancer agents. For instance, OATP1B3 has been shown to transport paclitaxel [9,10], docetaxel, actinomycin D, mitoxantrone, and SN-38 [11]. Methotrexate [12,13], SN-38 [14,15], flavopiridol [16], and the endothelin receptor antagonist atrasentan [17] may also be substrates of OATP1B1. OATP1A2 has been demonstrated to transport methotrexate [18] and imatinib [19]. Additionally, our previous studies [20] indicated that 5-fluorouracil and paclitaxel are likely transported by OATP1A2. The uptake of paclitaxel [21] and docetaxel [22] by OATP1A2 has been confirmed in humanized transgenic mice. Therefore, OATPs, especially the above-mentioned OATP1A2, OATP1B1, and OATP1B3, may serve as novel and potential therapeutic targets for chemotherapy [7].

The activity level of membrane transport proteins in target cells is often considered a rate-limiting factor in drug pharmacokinetics, determining the efficiency of drug delivery to tumors [23]. Previous studies have shown that radiation can impact the expression and function of both efflux and uptake transporters. For instance, it was found that irradiation increased the mRNA expression of P-glycoprotein (P-gp), multidrug resistance-associated protein (MRP), and lung resistance-related protein (LRP) in breast cancer cell lines. However, no changes in the mRNA levels of these three efflux transporters were observed in colon cancer cell lines. Conversely, the protein levels of P-gp, MRP, and LRP significantly increased after irradiation, which resulted in increased resistance to anti-tumor drugs such as cisplatin, doxorubicin, and bendamustine in irradiated colon cancer cells. These findings may help explain the occurrence of multidrug resistance following radiation treatment [24]. Additionally, previous research in our laboratory demonstrated that both X-ray [25] and carbon ion [20] irradiation induced the expression of OATP1A2 in the breast cancer cell line MCF7, particularly at low doses (0.5 Gy). This increase in OATP1A2 levels on the plasma membrane led to enhanced uptake of methotrexate [20,25], 5-fluorouracil, and paclitaxel [20]. Interestingly, the hormone-independent breast cancer cell line MDA-MB231 exhibited only a moderate response to carbon ion irradiation, showing minimal changes in OATP1A2 expression and drug absorption compared to MCF7 [20]. This suggests that different cell lines may respond variably to the same radiation treatment.

In the present study, the effect of high linear energy transfer (LET) carbon ions on the expression levels of OATP transporters in various cancer cell lines was investigated. It was shown that OATP1A2 was up-regulated only in the MCF7 breast cancer cell line and the HepG2 liver carcinoma cell line. The different responses may be partially attributed to the activation of the nuclear receptors farnesoid X receptor (FXR) and nuclear factor erythroid 2-related factor 2 (Nrf2) in the different cell lines. The interaction between FXR and Nrf2 with the upstream regulatory sequence of *SLCO1A2*, the gene that encodes OATP1A2, was analyzed. The role of these transcription factors in the carbon-ion induction of OATP1A2 was evaluated.

## 2. Materials and Methods

### 2.1. Materials

Dulbecco’s Modified Eagle’s Medium (DMEM), M-MLV reverse transcriptase, Platinum^®^ Taq DNA Polymerase, TRIzol^®^ reagent were sourced from Thermo Fisher Scientific (Waltham, MA, USA). Bio-Rad (Hercules, CA, USA) provided reagents for quantitative PCR. All other reagents, except where indicated, were purchased from Sigma (St. Louis, MO, USA).

### 2.2. Cell Culture and Irradiation Procedure

Human colon carcinoma cell line HCT116 (ATCC #CCL-247, RRID CVCL_0291), liver carcinoma cell line HepG2 (ATCC #HB-8065, RRID CVCL_0027), lung carcinoma cell line A549 (ATCC #CCL-185, RRID CVCL_0023), and breast cancer cell line MCF7 (ATCC #HTB-22, RRID CVCL_0031) were all purchased from the American Type Culture Collection (Manassas, VA, USA) and grown at 37 °C and 5% CO_2_ in DMEM supplemented with 10% fetal bovine serum. Confluent cells in 60 mm dishes were exposed to the carbon ion beam that was accelerated by the Heavy Ion Research Facility in Lanzhou (HIRFL), China. Physical characteristics, irradiation procedures, and dosimetry calculation of the HIRFL beam-delivery system were described as before [20]. Cells were irradiated by 135 MeV/nucleon carbon ions, whose LET in water at the sample position was measured to be 21 keV/μm with the indicated dosage at a dose rate of 1 Gy/min. Cells were then collected at different time points and subjected to different post-irradiation analyses.

### 2.3. RNA Isolation and RT-PCR

Total RNA was isolated from cells using the TRIzol^®^ reagent. An equal amount of RNA was used for the reverse transcription reaction, resulting in cDNA that was analyzed using real-time quantitative polymerase chain reaction (qPCR). The SYBR Green fluorescent dye methodology was employed along with an ABI Prism 7000 detector (Applied Biosystems, Foster City, CA, USA), as previously described [25]. Gene-specific primers for the OATP coding genes (*SLCO1A2*, *SLCO1B1*, and *SLCO1B3*) and nuclear receptors were designed using Primer3 software (Whitehead Institute for Biomedical Research, Cambridge, MA, USA) (Table 1). Dissociation curves of the PCR products were analyzed at the end of each qPCR reaction to confirm the specificity of each gene amplification. Relative quantification of the target mRNA levels was calculated by normalizing the total amount of cDNA tested to a *GAPDH* endogenous reference.

### 2.4. Construction of Plasmid DNA Containing Different Fragments of the Upstream Regulatory Sequence of SLCO1A2

Plasmids containing various fragments of the upstream regulatory sequence of *SLCO1A2* were constructed using the Omega-PCR technique [26]. The -293~+73 bp fragment was generated previously in our laboratory [27] and served as the template for mutagenesis. The QuikChange Lightning Site-Directed Mutagenesis Kit (Agilent, Santa Clara, CA, USA) was used for site-directed mutagenesis. All mutants were subjected to full-length sequencing to confirm correct sequences.

### 2.5. Transfection of Plasmid Constructs into Cells

Confluent HepG2 and MCF7 cells cultured in 48-well plates were transfected with the DNA plasmids using the LipofectAMINE 2000 reagent (Thermo Fisher Scientific), following the manufacturer’s instructions. The transfected cells were incubated at 37 °C for 24 h before undergoing further analyses.

### 2.6. Luciferase Assay

Luciferase activity was measured with the Dual-Luciferase Reporter Assay System (Promega, Madison, WI, USA) according to the manufacturer’s instructions. Briefly, cells in the 48-well plates were lysed with passive lysis buffer for 20 min at room temperature. An aliquot of the lysate (20 μL) was added to 100 μL of Luciferase Assay Reagent II, and the resulting signal was read on a GloMax^®^-Multi Jr Single Tube Multimode Reader (Promega). Firefly luminescence was subsequently quenched, and Renilla luciferase was activated concurrently by addition of 100 μL of Stop & Glo^®^ Reagent.

### 2.7. Electrophoretic Mobility Shift Assay

Biotin labeling of oligonucleotides containing the putative nuclear receptor binding sequence was carried out with the EMSA Biotin-labeling kit from Beyotime Biotechnology Inc. (Jiangsu, China), following the manufacturer’s instructions. Nuclear extracts (3 μg) and labeled oligonucleotide (100 nM) were combined using the EMSA/Gel-shift kit (Beyotime). After incubating for 30 min at room temperature, loading buffer was added, and the samples were loaded onto a non-denaturing polyacrylamide gel. Electrophoresis was performed for 1.5 h at 4 °C at 90 V in 0.5× Tris/Borate/EDTA buffer, followed by transfer to a positively charged nitrocellulose membrane. The membrane was cross-linked with UV light at 254 nm, blocked, and then incubated with streptavidin-HRP conjugate at room temperature for 30 min. The signals were detected with the Immobilon Western Chemiluminescent HRP Substrate from Merck Millipore (Billerica, MA, USA).

### 2.8. Chromatin Immunoprecipitation

ChIP assay kit from Beyotime Biotechnology Inc. was utilized for chromatin immunoprecipitation (ChIP) following the previously established protocol [27]. Briefly, 1% formaldehyde was applied to cells for cross-linking for 10 min at room temperature; the reaction was then quenched with glycine. Cells were lysed with SDS lysis buffer (50 mM Tris-HCl, pH 8.0; 10 mM EDTA; 1% SDS with protease inhibitors) and subjected to sonication using a Branson S450-D digital sonifier (Branson Ultrasonic, Danbury, CT, USA). After being pre-cleaned with Protein G agarose and salmon sperm DNA, the samples were subjected to centrifugation to eliminate the agarose beads. Then antibody (1:100 dilution) was added to the supernatant and incubated at 4 °C overnight. Protein G agarose/salmon sperm DNA was added to pull down the antibody-bound proteins, after which the beads were washed with a high-salt wash buffer (0.1% SDS, 0.1% Triton X-100, 500 mM NaCl, 2 mM EDTA, 20 mM Tris-HCl, pH 8.0) and TE buffer, and the protein-DNA complexes were released with the elution buffer (0.1% SDS, 0.1 M NaHCO_3_, pH 8.0). The DNA was purified using the High Pure PCR Product Purification Kit (Roche Applied Science, Penzberg, Germany) and subsequently analyzed by qPCR using the following primers: FXR (ER10): Forward: 5′-GGTACTATTTGTTTTGG CTAG-3′ and Reverse: 5′-TTAGTGACTCTTTAGTGAGAT- 3′. Nrf2 (ARE1): Forward: 5′-TCTTCTTCCAACGTGGAAAC-3′ and Reverse: 5′-GTCCAAGGACTGACTTGATG-3′; Nrf2 (ARE2): Forward: 5′-CAACACAGAGCTAT GACCA G-3′ and Reverse 5′-TCCCTAACTCTTTCTCAGCA-3′.

### 2.9. Survival Assay for Cells Co-Treated with Modulators, Radiation and Anti-Tumor Drugs

Cells were seeded in 96-well plates at a concentration of 5000 cells per well and allowed to incubate for 18 to 20 h before irradiation. Different modulators (at a concentration of 10 μM) were added 1 h prior to irradiation, and the anti-tumor drug methotrexate (10 μM) was administered 1 h after irradiation. The survival fraction of the cells was measured using the WST-1 Cell Proliferation and Cytotoxicity Assay Kit (Beyotime Biotechnology Inc.) as previously described [20]. Twenty-four hours after irradiation treatment, WST-1 (1/10 dilution) was added to each well, and the cells were incubated for 1 h at room temperature. The absorbance was then measured at 450 nm using a Tecan Infinite M200PRO plate reader (Männedorf, Switzerland).

### 2.10. Data Analysis

Student’s *t*-test was utilized for statistical analysis when comparing two groups of samples. One-way analysis of variance (ANOVA) followed by Bonferroni or Tukey’s post hoc test was employed for comparisons involving more than two groups. Data were considered significant when *p* < 0.05. All analyses were performed using GraphPad Prism version 8.0.2.

## 3. Results

### 3.1. Carbon Ion Irradiation Induced OATP1A2 Expression in HepG2 and MCF7 Cells

Since our previous study found that low-dose irradiation (IR) induces OATP expression more efficiently [20], we first irradiated different cell lines with 0.5 Gy of carbon ions. As shown in Table 2, IR treatment only caused a marginal change or even a reduction in OATP1A2, 1B1, and 1B3 expression in A549 and HCT116 cells when compared to the non-IR control at the same time points. On the other hand, although OATP1B1 and/or OATP1B3 exhibited minor changes in MCF7 and HepG2, the expression of OATP1A2 showed a significant increase in both cell lines after IR treatment. Additionally, the induction of OATP1A2 in both cell lines peaked at 6 h after IR treatment.

### 3.2. Carbon Ion Irradiation Induced the Expression of FXR

Nuclear receptors (NRs) have been widely recognized as important for the regulation of the expression of OATPs [28]. The major nuclear receptors that regulate the drug-related transporters include pregnane X receptor (PXR), constitutive androstane receptor (CAR), FXR, and liver X receptor (LXR). Therefore, we further analyzed the expression of these NRs after irradiation. As shown in Table 3, most of the NRs showed a marginal change after IR, while FXR started to show an increase at 3 h post-IR and exhibited more than 2-fold up-regulation in MCF7 and HepG2 cells 6 h after IR treatment. FXR and PXR in HCT116 also showed a significant elevation at 6 and 20 h after IR, respectively. CAR, however, was below the detectable level or exhibited unstable detection in all cell lines.

### 3.3. FXR Interacts with SLCO1A2 Upstream Regulatory Sequence

**Figure 1:**
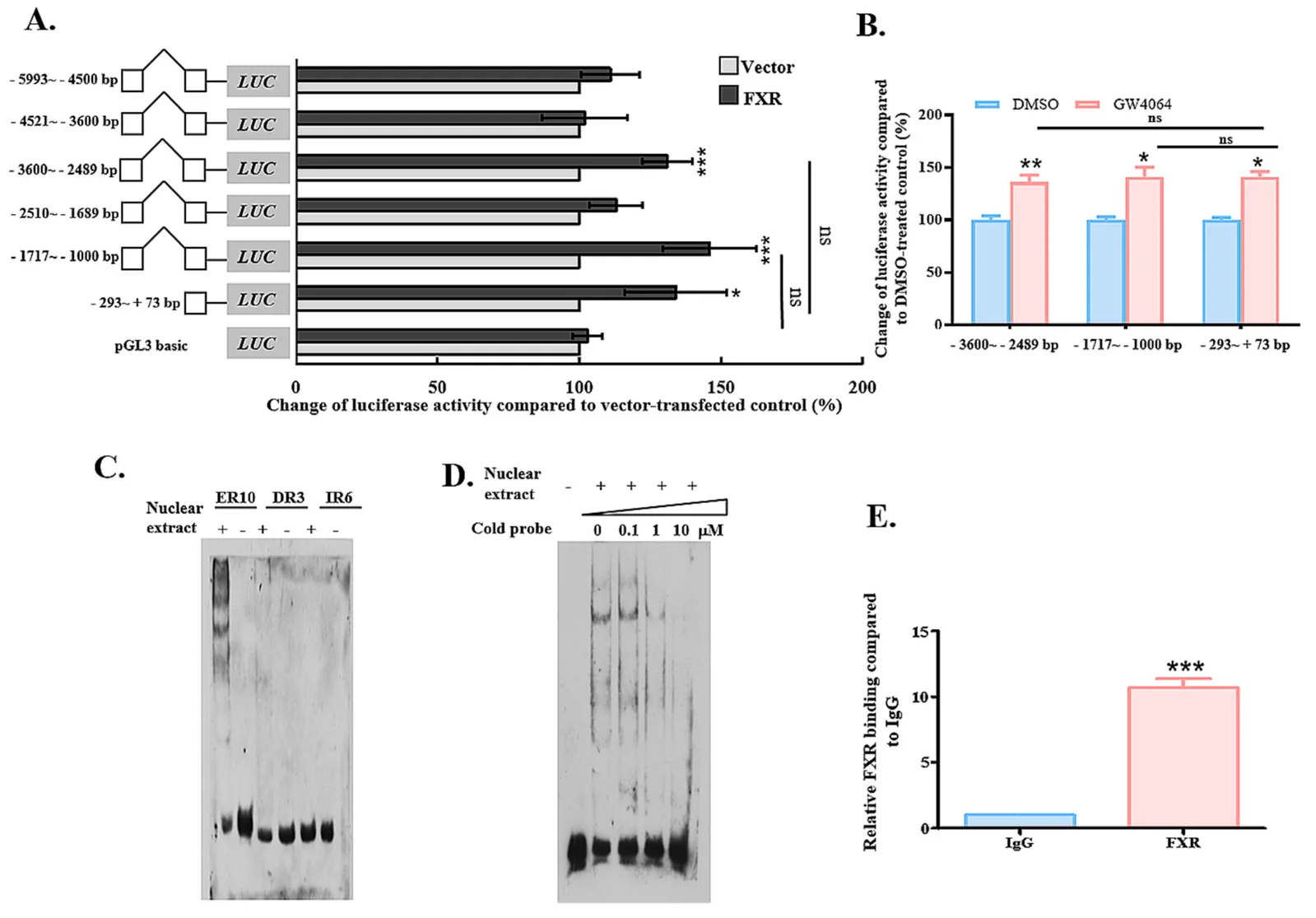
FXR interacts with *SLCO1A2* upstream sequences in HepG2 cells. (**A**) FXR overexpression effect on the luciferase activity of *SLCO1A2* upstream sequences. (**B**) Influence of GW4064 on different upstream fragments of *SLCO1A2*. Cells were co-transfected with the FXR expression plasmid together with plasmids carrying various upstream regulatory fragments of *SLCO1A2*, or cells transfected with the *SLCO1A2* upstream constructs were exposed to 10 μM GW4064 for 24 h. Luciferase reporter activity was subsequently determined. (**C**,**D**). EMSA was utilized to assess the binding of nuclear proteins to the predicted FXR-responsive ER10 element following the procedure described in the Materials and Methods section. For the competition assay (**D**), unlabeled probes were added at 1-, 10-, or 100-fold molar excess relative to the labeled probe (100 nM) to evaluate binding specificity. (**E**) ChIP was conducted to verify the association of FXR with the predicted binding region, as described in the Materials and Methods section. Rabbit IgG (IgG) was used as a negative control for nonspecific immunoprecipitation. Luciferase activity was normalized to the corresponding vector-transfected or DMSO-treated control, which was defined as 100% within each experiment. Data are presented as mean ± SEM from three independent experiments (*n* = 3). Student’s *t*-test was utilized for the statistical analysis. Asterisks indicate values significantly different (* *p* < 0.05, ** *p* < 0.01, *** *p* < 0.001) from those of the empty vector-transfected or DMSO-treated control. ns, not statistically significant.

Since the FXR level was elevated in both MCF7 and HepG2 cells, we speculated that this transcription factor may be involved in the induction of OATP1A2 by heavy ion IR. When the upstream 6 kb untranslated region of *SLCO1A2* was analyzed [29], it was found that there are five possible FXR binding sites predicted by the computer algorithm NUBIscan (Table 4). The corresponding segments that contain different binding sites were then generated. Since *SLCO1A2* has a minimal promoter region within 91 bp upstream to the transcription initiation site [30], the segment (construct −293~+73 bp) that contains the region was included in all the luciferase expression constructs. It was found that the overexpression of FXR significantly increased luciferase activity of the constructs of −293~73 bp, −1717~−1000 bp, and −3600~−2489 bp, which contain the putative FXR binding domains of ER-10 (all three constructs), IR-6 (both −293~73 bp and −1717~−1000 bp constructs), and DR-3 (−3600~−2489 bp construct) (Figure 1A and Table 4). Similarly, the treatment with FXR agonist GW4064 significantly increased the luciferase activity of these three constructs (Figure 1B). However, the elevation of −1717~−1000 bp and −3600~−2489 bp after overexpressing FXR or GW4064 treatment was not significantly higher than the induction of construct −293~73 bp, suggesting that the increased luciferase activity of both −1717~−1000 bp and −3600~−2489 bp may be merely due to the induction of the −293~73 bp fragment, which is included in the former two constructs. Indeed, EMSA analysis revealed that nuclear extracts from HepG2 cells only specifically interacted with the oligo containing ER-10 but not those derived from IR-6 or DR-3 (Figure 1C,D). The interaction between FXR and ER-10 was also confirmed by ChIP (Figure 1E).

### 3.4. Nrf2 Interacts with SLCO1A2 Upstream Regulatory Sequence

**Figure 2:**
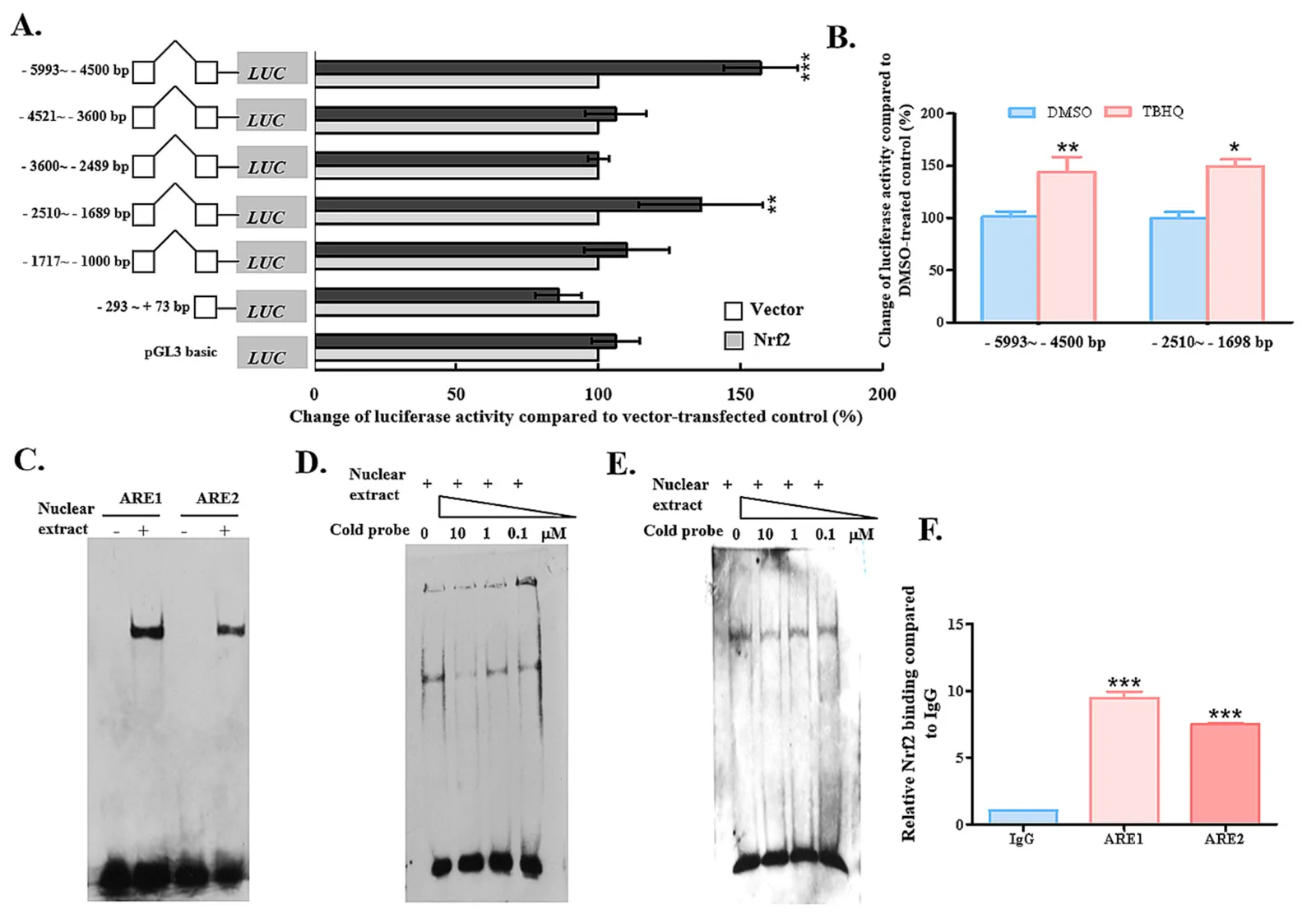
Interaction of Nrf2 with *SLCO1A2* upstream sequences in HepG2 cells. (**A**) Influence of Nrf2 overexpression on the luciferase activity of *SLCO1A2* upstream sequences. (**B**) Response of the upstream fragments of *SLCO1A2* to TBHQ treatment. HepG2 cells were either co-transfected with the Nrf2 expression plasmid and reporter constructs containing different *SLCO1A2* upstream regions, or transfected solely with the reporter constructs followed by exposure to 10 μM TBHQ for 24 h. Luciferase activity was then determined. (**C**) Detection of nuclear protein binding to the predicted Nrf2-responsive elements by EMSA, which was performed according to the procedure described previously. Competitive assay for ARE1 (**D**) and ARE (**E**) was carried out by adding 1-, 10-, or 100-fold molar excess of unlabeled oligonucleotides to the labeled probe (100 nM). (**F**) ChIP assay examining the association of Nrf2 with the predicted regulatory sequences, as described in the Materials and Methods section. Rabbit IgG (IgG) was included as the nonspecific binding control. Luciferase activity was normalized to the corresponding vector-transfected or DMSO-treated control, which was defined as 100% within each experiment. Data are presented as mean ± SEM from three independent experiments (*n* = 3). Student’s *t*-test was utilized for statistical analysis when comparing two groups of samples. One-way analysis of variance (ANOVA) followed by Bonferroni post hoc test was employed for comparisons involving more than two groups. Asterisks indicate values significantly different (* *p* < 0.05, ** *p* < 0.01, *** *p* < 0.001) from those of the empty vector-transfected or DMSO-treated control.

Nrf2 is a transcription factor that has been found to regulate the expression of membrane proteins, especially under stress that generates reactive oxygen species (ROS). We therefore investigated whether Nrf2 also plays a role in the induction of OATP1A2 after IR. NUBIscan analysis predicted two possible binding sites in the upstream regulatory sequence of *SLCO1A2* (Table 4). Correspondingly, overexpression of Nrf2 or treatment with the Nrf2 agonist TBHQ significantly increased the luciferase activity of the constructs −2510~−1698 bp and −5993~−4500 bp (Figure 2A,B). Additionally, EMSA (Figure 2C–E) and ChIP (Figure 2F) further confirmed that nuclear extracts from HepG2 cells specifically interacted with oligos derived from these two predicted sequences.

**Figure 3:**
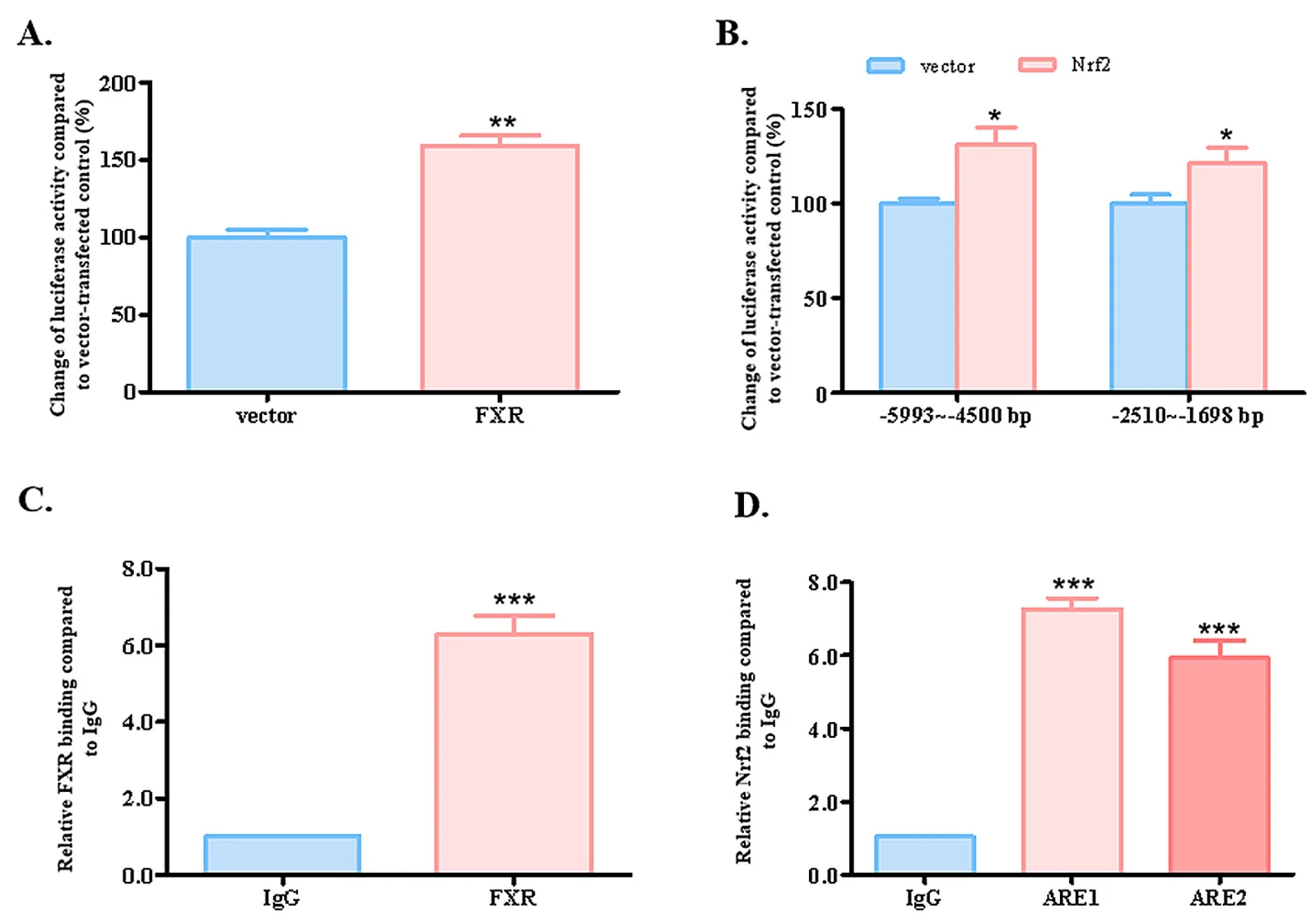
FXR and Nrf2 also interact with the *SLCO1A2* upstream sequences in MCF7 cells. Luciferase activity of the corresponding fragments was also affected in MCF7 cells transfected with FXR (**A**) or Nrf2 (**B**) overexpressing constructs. ChIP analysis was performed to assess the interaction of FXR (**C**) and Nrf2 (**D**) with the putative binding sequence in MCF7 cells. Relative luciferase activity was expressed as a percentage of the vector-transfected control, which was set as 100% for each set of experiments. The results were expressed as mean ± SEM (*n* = 3) with data from three independent experiments. Student’s *t*-test was utilized for statistical analysis when comparing two groups of samples. One-way analysis of variance (ANOVA) followed by Bonferroni post hoc test was employed for comparisons involving more than two groups. Asterisks indicate values significantly different (* *p* < 0.05, ** *p* < 0.01, *** *p* < 0.001) from those of the empty vector-transfected or Ig-G control.

To evaluate whether FXR and Nrf2 also exhibit a regulatory role in upregulating OATP1A2 in MCF7, we overexpressed the transcription factors and evaluated the induction effect. As shown in Figure 3, the overexpression of FXR and Nrf2 both induced luciferase activity of the corresponding constructs in MCF7 cells (Figure 3A,B). ChIP analysis also showed that these two transcription factors interact with the corresponding upstream sequences of *SLCO1A2* in MCF7 cells (Figure 3C,D).

**Figure 4:**
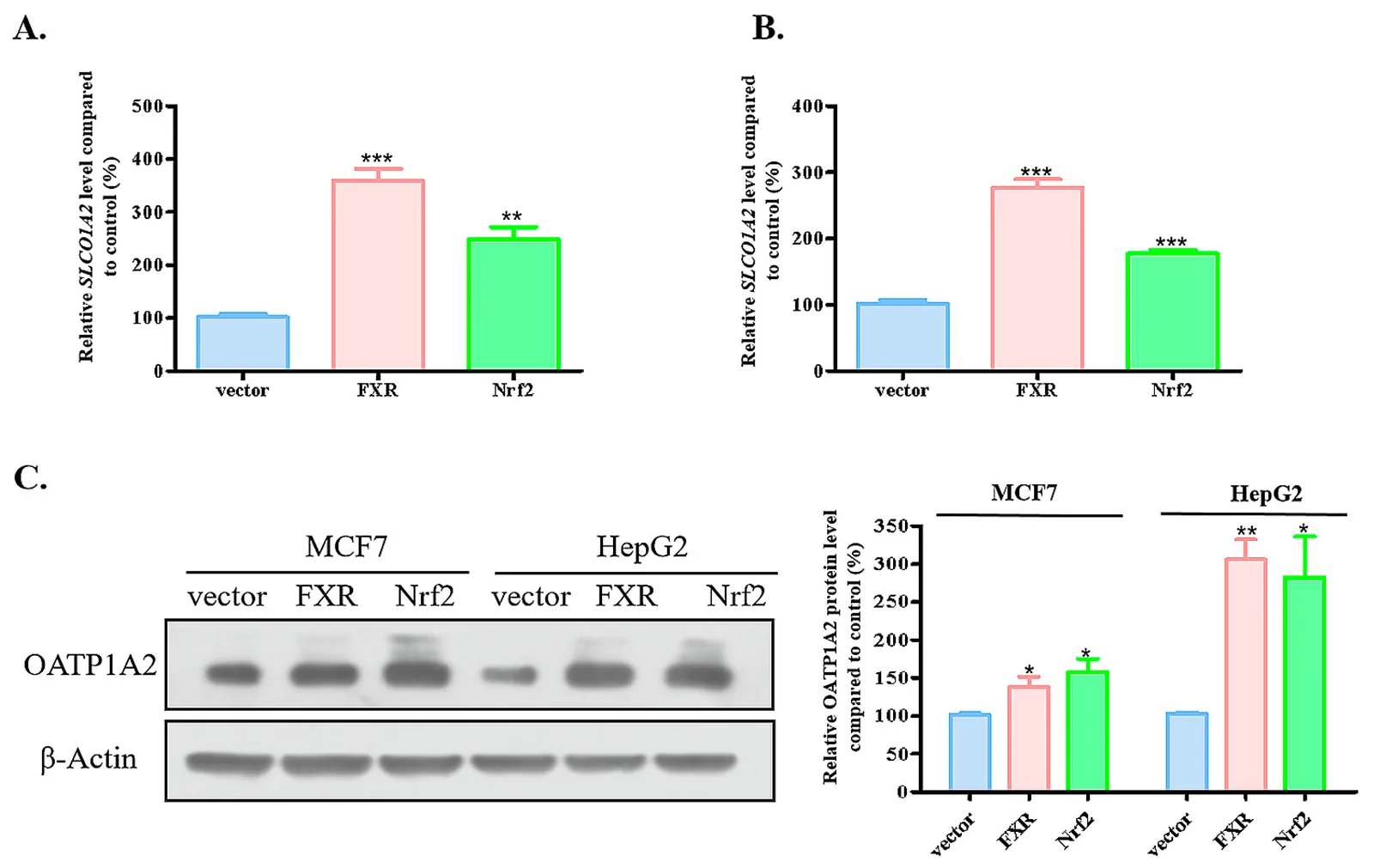
FXR and Nrf2-induced *SLCO1A2* expression in HepG2 and MCF7 cells. mRNA level of OATP1A2 in HepG2 (**A**) and MCF7 (**B**) cells overexpressing FXR or Nrf2. Cells were transfected with FXR or Nrf2, then RNA was collected and analyzed with qPCR. (**C**) Protein level of OATP1A2 in HepG2 and MCF7 cells overexpressing FXR or Nrf2. Proteins were collected and analyzed with Western blotting (Figure S1). Representative blot was shown. Protein expression was analyzed with Image J and the OATP1A2 protein level was normalized to the β-actin level from the same sample in each set of experiment. Relative mRNA and protein levels were expressed as a percentage of the vector-transfected control, which was set as 100% for each set of experiments. The results were expressed as mean ± SEM (*n* = 3) with data from three independent experiments. One-way analysis of variance (ANOVA) followed by Bonferroni post hoc test was employed for the comparisons. Asterisks indicate values significantly different (* *p* < 0.05, ** *p* < 0.01, *** *p* < 0.001) from those of the empty vector-transfected control.

Furthermore, to confirm the effect of FXR and Nrf2 on the expression of OATP1A2, we overexpressed the transcription factors in HepG2 and MCF7 cells. As shown in Figure 4, OATP1A2 was significantly upregulated at both the mRNA and protein levels in HepG2 and MCF7 cells.

### 3.5. OATP1A2 Induction by Carbon Ion Irradiation Is Mediated by FXR and Nrf2

**Figure 5:**
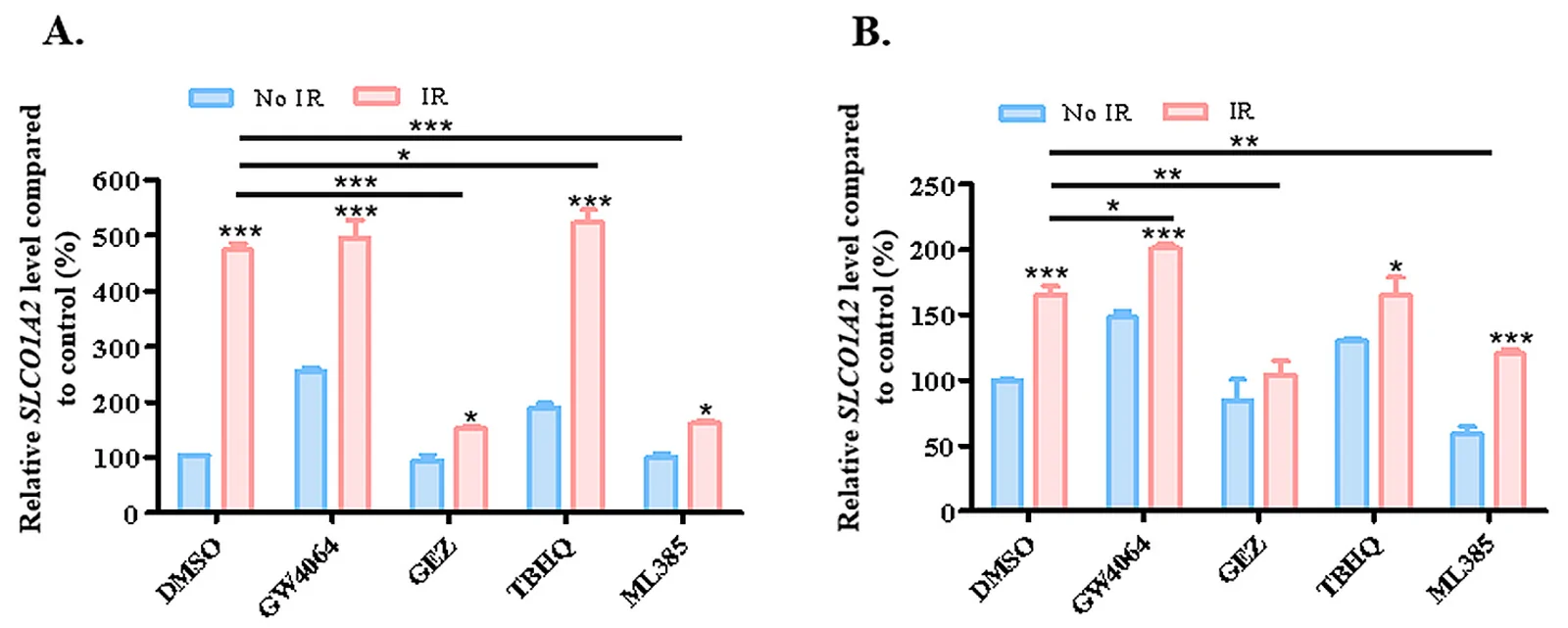
Modulators of FXR/Nrf2 affected the induction of OATP1A2 by IR. (**A**) mRNA level of OATP1A2 after carbon ion IR with treatments of different FXR/Nrf2 modulators in HepG2 cells. (**B**) mRNA level of OATP1A2 after carbon ion IR with treatments of different FXR/Nrf2 modulators in MCF7 cells. Cells were pre-treated with different modulators (10 μM) for 1 h before irradiation. The samples were collected 6 h after irradiation and subjected to qPCR analysis. Relative mRNA levels were normalized to the corresponding DMSO-treated, non-irradiated control collected at the same time point, which was set as 100% for each set of experiments. The results were expressed as mean ± SEM (*n* = 3) with data from three independent experiments. One-way analysis of variance (ANOVA) followed by Bonferroni post hoc test was employed for the statistical analysis. Asterisks indicate values significantly different (* *p* < 0.05, ** *p* < 0.01, *** *p* < 0.001) from those of the non-irradiated or DMSO-treated control.

To investigate whether the induction of OATP1A2 by IR is mediated by FXR and Nrf2, we treated cells with agonists of FXR (GW4064) and Nrf2 (TBHQ), as well as inhibitors of these transcription factors, i.e., guggulsterone E&Z (GEZ) for FXR and ML385 for Nrf2, and analyzed the effect of carbon ion on the expression of OATP1A2. As shown in Figure 5, inhibition of FXR and Nrf2 both attenuated the induction of OATP1A2 by IR treatment when compared to the DMSO-treated control. On the other hand, elevated activity of FXR and Nrf2 significantly enhanced the expression of OATP1A2 after IR, suggesting that the alteration of FXR and Nrf2 indeed affects the response of OATP1A2 in HepG2 and MCF7 cells to carbon ions.

**Figure 6:**
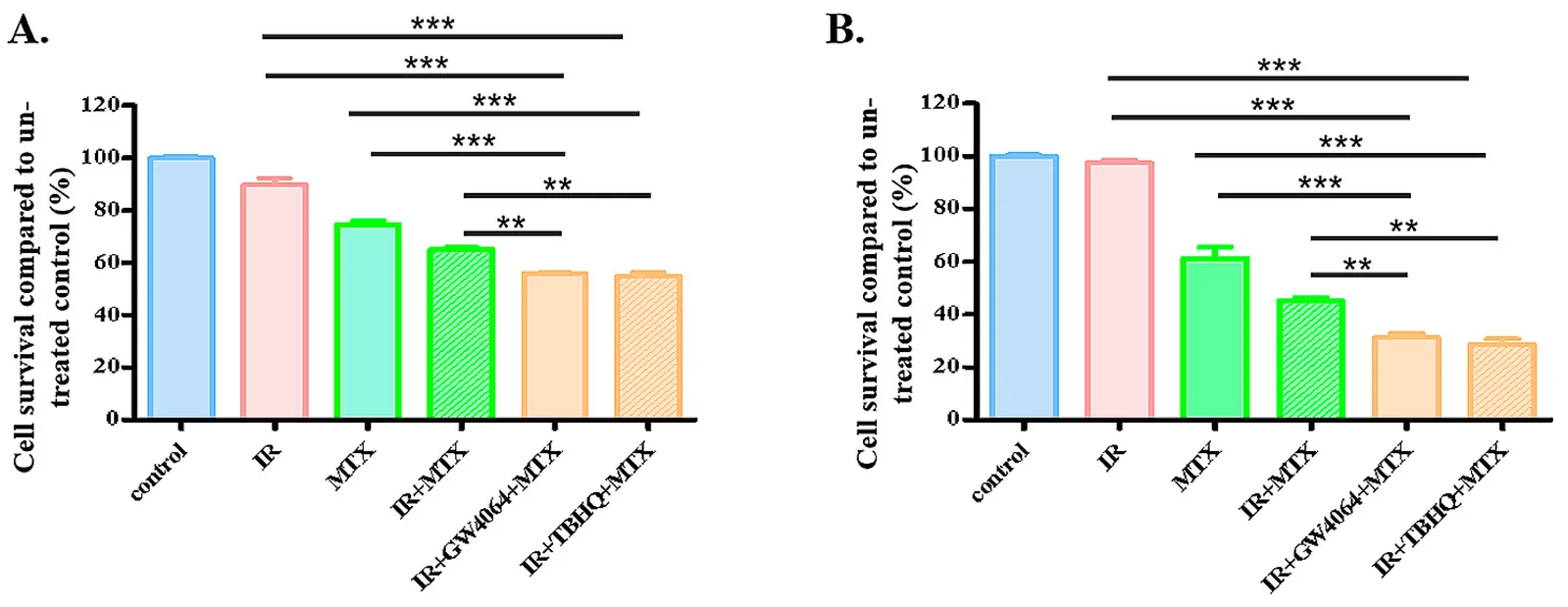
Induction of FXR/Nrf2 increased the IR and drug co-treatment effect on cells. (**A**) Combined effect of FXR/Nrf2 modulators and methotrexate on irradiated HepG2 cells. (**B**) Combined effect of FXR/Nrf2 modulators and methotrexate on irradiated MCF7 cells. Cells were treated with GW4064 or TBHQ 1 h before IR, while methotrexate was added 1 h after irradiation. Cell survival fraction was measured with the WST-1 assay 24 h after irradiation and normalized to the corresponding DMSO-treated, non-irradiated control collected at the same time point, which was set as 100% for each set of experiments. The results were expressed as mean ± SEM (*n* = 3) with data from three independent experiments. One-way analysis of variance (ANOVA) followed by Tukey’s post hoc test was applied for the comparisons among independent groups. The overall group effect was statistically significant for both Figure 6A (F(5, 12) = 205.4, *p* < 0.0001) and Figure 6B (F(5, 12) = 208.1, *p* < 0.0001). Asterisks indicate values significantly different (** *p* < 0.01, *** *p* < 0.001) between groups.

Moreover, methotrexate (MTX), an anti-tumor drug that is a substrate of OATP1A2 [18], exhibited enhanced cytotoxicity in both HepG2 and MCF7 cells treated with FXR and Nrf2 agonists and IR (Figure 6).

## 4. Discussion

Drug transporters are important determinants of the accumulation of therapeutic agents within cells and hence serve essential roles in the therapeutic efficacy of various medications, including many anti-neoplastic drugs. Our previous studies demonstrated that low-dose X-ray and carbon ion led to enhanced expression of OATP1A2, a member of the eminent drug uptake transporter family OATP, in the estrogen-receptor-positive breast cancer MCF7 cells, while in the meantime causing marginal change in the expression of the efflux transporters P-gp and BCRP, which results in increased accumulation of anti-tumor drugs and killing effect on cancer cells [20,25]. In the present study, it was found that the low-dose heavy-ion effect on OATP1A2 is cell type-dependent, and the transcription factors FXR and Nrf2 are involved in the process.

Four cancer cell lines were evaluated in our current study, and it was found that only HepG2 and MCF7 exhibited upregulation of OATP1A2 during the tested time period. The enhancement of OATP1A2 in MCF7 cells is consistent with our previous report [20]. On the other hand, it was revealed that HCT116, a colon cancer cell line, and A549, a cell line derived from lung adenocarcinoma, exhibited marginal changes in OATP1A2. We postulate that such differences may be due to the different cellular environment within these cell lines. Studies have shown that steroid hormones can enhance the growth of HepG2 [31] and MCF7 [32], implicating an induction effect on these cells. OATP family members, which are considered to be major uptake transporters for steroid hormones, have been demonstrated to be regulated by the increased level of hormones in certain cancer cell types [33]. Whether the hormone contents within these cells affect their responses to irradiation warrants further investigation.

The expression of OATPs has been demonstrated to be regulated by NRs. We therefore first evaluated the changes in several commonly found regulatory NRs after IR. Previous studies indicated that PXR, an NR that regulates various metabolizing enzymes and transporters, affects the expression of OATP1A2 [34]. However, our present study found that the expression of PXR exhibited no change after heavy ion IR. On the other hand, the level of FXR, a bile acid-activated NR that is mainly expressed in the liver and intestine, was found to be upregulated at a timeline that is comparable to OATP1A2 induction. Further analysis revealed that FXR specifically binds to the upstream sequence of *SLCO1A2*, validating the regulatory effect of the NR on OATP1A2 expression. However, it should be noted that FXR expression was also induced in HCT116, which exhibited no significant change in OATP1A2 at the test time period. Moreover, PXR level was significantly up-regulated in this colon cancer cell line, albeit at a later time point (20 h post-IR). Therefore, it is possible that other factors within the specific cellular environment of these cancer cells are also involved in the regulation of OATP1A2. For example, microRNAs may affect the mRNA stability of OATP1A2 at the post-transcriptional level. The binding sites for miR-324, together with miR-1913, were suggested at the 3′UTR of *SLCO1A2* using bioinformatics tools [35], while previous studies have reported elevated miR-324-3p expression in colorectal cancer (CRC) cells, including HCT116 cells [36]. Therefore, the relatively high abundance of miR-324-3p in CRC cells may potentially attenuate FXR-induced *SLCO1A2* expression at the post-transcriptional level. Additionally, several microRNAs, including miR-21, miR-31, and miR-135b, have been demonstrated to be specifically up-regulated in CRC patients and proposed to serve as diagnostic and prognostic markers for CRC [37]. Although there have not been reports concerning the regulation of *SLCO1A2* by these microRNAs, they may also contribute to the limited induction of endogenous *SLCO1A2* in HCT116 cells after IR. A transcriptome analysis for microRNA profile changes after heavy ion IR in different cell lines is currently underway in our laboratory to clarify this issue. In addition, there have been reports demonstrating that transporter proteins may have different transcriptional start site variants in different kinds of cells, which lead to differentiated regulatory mechanisms. For example, OATP2B1, another OATP family member, was shown to have a dominant variant, OATP2B1-1e, in the liver that is regulated by hepatocyte nuclear factor 4 alpha (HNF4α). However, OATP2B1-1b, another variant that is ubiquitously expressed in other tissues, exhibits no response to HNF4α [38]. Thus, it is also possible that a different variant of *SLCO1A2* is present in HCT116 cells compared to that in MCF7 and HepG2 cells, resulting in a different FXR binding profile that leads to the lack of regulation by this transcription factor in HCT116 cells, although this possibility requires direct validation in HCT116 cells.

In addition to FXR, Nrf2 was also found to be responsible for the regulation of OATP1A2. Nrf2 is one of the master regulators in cellular antioxidant responses. Downstream genes regulated by Nrf2 include detoxification enzymes such as cytochrome P450 1B1, UDP glucuronosyltransferase 1A1, as well as efflux transporters such as ABCB6 [39]. A recent study in our lab found that heavy-ion IR induces nuclear accumulation of Nrf2, which in turn binds to the upstream sequence of zinc transporter ZIP14 and enhances the DNA damage repair capability of the IR cells [40], suggesting that the upregulation of Nrf2 is likely a common response of cells toward low-dose heavy-ion IR.

Furthermore, pre-treating HepG2 and MCF7 cells with FXR or Nrf2 agonists prior to IR and treatment with anti-neoplastic agents resulted in a significantly greater killing effect than simply applying methotrexate after IR. This implies that inducing FXR and Nrf2 may boost the accumulation of methotrexate following IR, ultimately leading to a more substantial impact on cell survival. However, whether the uptake of methotrexate by OATP1A2 indeed contributes to the enhanced cytotoxicity remains to be elucidated. Further studies are also needed to determine whether this response can be replicated in vivo.

It should also be noted that the roles of FXR and Nrf2 in regulating *SLCO1A2* expression and cell survival under IR were evaluated primarily using pharmacological modulators, with overexpression experiments providing supporting evidence. Because pharmacological agents can have off-target effects, caution should be exercised in the mechanistic interpretation of these data. Future studies employing knock-down/-out NR systems will be needed to more precisely define the roles of FXR and Nrf2 in the IR-induced regulation of *SLCO1A2*.

## 5. Conclusions

In summary, our present study demonstrated that the heavy ion induction of OATP1A2 is cell-type-specific. Both FXR and Nrf2 are involved in the upregulation of the transporter by IR, and the application of their agonists together with IR may be a novel strategy to enhance the cell-killing effect in combined chemoradiotherapy.

## Figures and Tables

**Table 1:** Primers for the quantitative PCR analysis of OATPs and NRs.

OATPs	Forward Primers	Reverse Primers
*SLCO1A2* (OATP1A2)	CTTTGAAATGTGGTGAGTCAG	AAGGCTGGAACAAAGCTTGA
*SLCO1B1* (OATP1B1)	ATCTTATTGGGAGTCATAACCA	GGATTATTTCCATCATAGGTCA
*SLCO1B3* (OATP1B3)	TCAAGTGGTATTAAAAAGCATAC	TTCACCCAAGTGTGCTGAGT
*NR2l2* (PXR)	GCTGTCCTACTGCTTGGAAGAC	CTGCATCAGCACATACTCCTCC
*NR1I3* (CAR)	ACCTGTGCAACTGAGTAAGG	ATGTCTGCGAAGTGTGTGAC
*NR1H4* (FXR)	CTGCGGTTGAAGCTATGTTC	CTGTAAGCAGAGCATACTCC
*NR1H3* (LXR)	CCATGAATGAGCTGCAACTC	AGGGCTTCCACATATGTGTG

**Table 2:** OATP expression in different cancer cells after carbon ion irradiation.

Time Post-Irradiation (h)		3	6	20
A549	*SLCO1A2*	0.97 ± 0.45	1.12 ± 0.19	0.38 ± 0.12
	*SLCO1B1*	N.D.	N.D.	N.D.
	*SLCO1B3*	1.35 ± 0.42	1.48 ± 0.41	0.55 ± 0.09
HCT116	*SLCO1A2*	0.82 ± 0.29	0.94 ± 0.10	0.69 ± 0.21
	*SLCO1B1*	0.19 ± 0.11	0.66 ± 0.34	0.29 ± 0.05
	*SLCO1B3*	0.52 ± 0.11	0.77 ± 0.36	0.84 ± 0.13
MCF7	*SLCO1A2*	1.25 ± 0.39	**2.45 ± 1.00**	0.73 ± 0.38
	*SLCO1B1*	1.17 ± 0.57	0.89 ± 0.25	0.96 ± 0.07
	*SLCO1B3*	N.D.	N.D.	N.D.
HepG2	*SLCO1A2*	1.69 ± 0.92	**5.27 ± 0.81**	1.40 ± 0.20
	*SLCO1B1*	0.88 ± 0.30	1.26 ± 0.16	0.66 ± 0.48
	*SLCO1B3*	0.98 ± 0.05	0.78 ± 0.24	1.17 ± 0.43

Cells were irradiated with 0.5 Gy carbon ions and collected at different time points for qPCR analysis. Expression level of OATPs was compared to the non-irradiated control collected at the same time point. Expression change greater than 2-fold is in bold. A Ct value of more than 35 was defined as the limit of detection, and samples with Ct > 35 was considered as not reliably detected (N.D.). The results are expressed as mean ± SEM (*n* = 3) with data from three independent experiments.

**Table 3:** Nuclear receptor expression in different cancer cells after carbon ion irradiation.

Time Post-Irradiation (h)		3	6	20
A549	*PXR*	1.51 ± 0.28	1.62 ± 0.77	0.67 ± 0.33
	*FXR*	1.11 ± 0.40	1.23 ± 0.40	0.75 ± 0.21
	*LXR*	1.51 ± 0.14	1.03 ± 0.25	0.58 ± 0.01
HCT116	*PXR*	0.46 ± 0.07	1.15 ± 0.30	**2.51 ± 0.16**
	*FXR*	1.41 ± 0.75	**2.47 ± 0.85**	1.69 ± 0.48
	*LXR*	1.02 ± 0.15	1.88 ± 0.83	1.12 ± 0.27
MCF7	*PXR*	0.92 ± 0.39	1.00 ± 0.54	0.99 ± 0.03
	*FXR*	1.34 ± 0.56	**2.10 ± 0.10**	1.26 ± 0.52
	*LXR*	1.02 ± 0.15	1.40 ± 0.77	1.12 ± 0.27
HepG2	*PXR*	1.07 ± 0.27	1.09 ± 0.09	0.97 ± 0.17
	*FXR*	1.66 ± 0.29	**2.59 ± 0.81**	1.15 ± 0.66
	*LXR*	0.90 ± 0.41	1.09 ± 0.10	0.84 ± 0.32

Cells were irradiated with 0.5 Gy carbon ions and collected at different time points for qPCR analysis. Expression level of NRs was compared to the non-irradiated control collected at the same time point. Expression change greater than 2-fold is in bold. The results are expressed as mean ± SEM (*n* = 3) with data from three independent experiments.

**Table 4:** Putative binding sites of FXR and Nrf2 in the 5′UTR region of *SLCO1A2*.

	Location of the Binding Site	Sequence of the Binding Site	Corresponding Luciferase Construct	Name of the Binding Site
FXR	−5023~−5002 bp	TGGTCATTTGTTTATCTGGCCT	−5993~−4500 bp	IR10
	−4352~−4337 bp	AGGTCACTTGAGAGCA	−4521~−3600 bp	DR4
	−3314~−3300 bp	AGGTAAAGGAGTTCA	−3600~−2489 bp	DR3
	−1195~−1178 bp	AGTTCAGAACAATAACCT	−1717~−1000 bp	IR6
	−84~−63 bp	TGACCATTATTTTGCAAGATCA	−293~73 bp	ER10
Nrf2	−5480~5470 bp	GTAACAAAGCA	−5993~−4500 bp	ARE1
	−2100~−2090 bp	GTGAGAAAGCA	−2510~−1698 bp	ARE2

IR: inverted repeats; DR: direct repeats; ER: everted repeats; ARE: antioxidant response element.

## Data Availability

The authors declare that all the data supporting the findings of this study are available within the paper.

## Supplementary Materials

The following supporting information can be downloaded at: https://www.mdpi.com/article/10.3390/biology15201886/s1, Figure S1: Original blots for Figure 4C.

## Abbreviations

CAR: constitutive androstane receptor; FXR: farnesoid X receptor; IR: irradiation; LET: linear energy transfer; LRP: lung resistance-related protein; LXR: liver X receptor; MRP: multidrug resistance-associated protein; Nrf2: nuclear factor erythroid 2-related factor 2; NR: nuclear receptor; OATP: organic anion transporting polypeptide; P-gp: P-glycoprotein; PXR: pregnane X receptor.
